# Nano vaccines for *T. gondii* Ribosomal P2 Protein With Nanomaterials as a Promising DNA Vaccine Against Toxoplasmosis

**DOI:** 10.3389/fimmu.2022.839489

**Published:** 2022-02-21

**Authors:** ZhengQing Yu, Ke He, WanDi Cao, Muhammad Tahir Aleem, RuoFeng Yan, LiXin Xu, XiaoKai Song, XiangRui Li

**Affiliations:** MOE Joint International Research Laboratory of Animal Health and Food Safety, College of Veterinary Medicine, Nanjing Agricultural University, Nanjing, China

**Keywords:** *Toxoplasma gondii*, ribosomal P2 protein, PLGA, chitosan, immunoprotection

## Abstract

Caused by *Toxoplasma gondii*, toxoplasmosis has aroused great threats to public health around the world. So far, no effective vaccine or drug is commercially available, and the demands for a safe and effective therapeutic strategy have become more and more urgent. In the current study, we constructed a DNA vaccine encoding *T. gondii* ribosomal P2 protein (TgP2) and denoted as TgP2-pVAX1 plasmid. To improve the immunoprotection, nanomaterial poly-lactic-*co*-glycolic acid (PLGA) and chitosan were used as the delivery vehicle to construct TgP2-pVAX1/PLGA and TgP2-pVAX1/CS nanospheres. Before vaccinations in BALB/c mice, TgP2-pVAX1 plasmids were transiently transfected into Human Embryonic Kidney (HEK) 293-T cells, and the expression of the eukaryotic plasmids was detected by laser confocal microscopy and Western blotting. Then the immunoprotection of naked DNA plasmids and their two nano-encapsulations were evaluated in the laboratory animal model. According to the investigations of antibody, cytokine, dendritic cell (DC) maturation, molecule expression, splenocyte proliferation, and T lymphocyte proportion, TgP2-pVAX1 plasmid delivered by two types of nanospheres could elicit a mixed Th1/Th2 immune response and Th1 immunity as the dominant. In addition, TgP2-pVAX1/PLGA and TgP2-pVAX1/CS nanospheres have great advantages in enhancing immunity against a lethal dose of *T. gondii* RH strain challenge. All these results suggested that TgP2-pVAX1 plasmids delivered by PLGA or chitosan nanomaterial could be promising vaccines in resisting toxoplasmosis and deserve further investigations and applications.

## Introduction

Widely distributed around the world, *Toxoplasma gondii* is an obligate intracellular parasite that can infect almost all mammals including human beings (1, 2). The invasion of *T. gondii* in newborns can lead to congenital defects, while the occurrence in immunocompromised individuals can cause pneumonia, encephalitis, even death (3, 4). In addition, toxoplasmosis in livestock can result in economic losses, especially in sheep and pigs (1). Chemical treatments mainly including pyrimethamine (PYR) and sulfadiazine (SDZ) are effective against *T. gondii* bradyzoites (5, 6), but their side effects cannot be ignored (7). Currently, therapeutic strategies to completely eradicate *T. gondii* are still unavailable (8). Ovilis Toxovax^®^ (Intervet Inc., Angers, France) so far has been the only *T. gondii* vaccine commercially available for abortion prevention in goats and sheep (9), but the immunoprotection of tissue cyst formation has been questioned by Food Standards Agency (10). In short, considering drug residues in animal products and the drug resistance in parasites (11), the development of effective vaccines against toxoplasmosis is a valued and urgent priority.

Safe and reliable vaccines against *T. gondii* may be the approach to control the infection (12). In recent years, great achievements have been made in the identification of vaccine candidates against *T. gondii* (13), and numerous vaccine candidates have been proved to be effective in resisting toxoplasmosis (14, 15). However, these tested vaccines cannot generate full protection against *T. gondii* in animals. The ability of *T. gondii* to cross the blood–brain barrier and the capability of developing a continuous infection in the central nervous system are a challenge for drug therapy due to the inaccessibility of chemical components and made the control of toxoplasmosis in humans and animals difficult (16). After a successful invasion, *T. gondii* could quickly develop into tachyzoites, which define the acute infection, leading to serious consequences in the host. As a ribosomal stalk protein, *T. gondii* ribosomal P2 protein (TgP2) plays an important role in *T. gondii* survival (17), and it has been proved to localize to the surface of tachyzoites (18). Such properties create a favorable condition for the host immunity to resist. Recent research indicated that ribosomal *Plasmodium falciparum* ribosomal P2 protein (PfP2) was immunogenic in mouse models (19), and PfP2 was highly homologous to TgP2 (about 70% similarity). In addition, identified by murine macrophages *in vitro*, recombinant TgP2 obtained from a prokaryotic expression system could induce immunoprotection against *T. gondii* infections *in vivo* (20). These observations suggest an important function of TgP2 in *T. gondii* invasion and survival, and the construction of *T. gondii* vaccines targeting the TgP2 looks rational in generating immunity against toxoplasmosis.

Previous studies have developed numerous vaccination strategies against toxoplasmosis, mainly including attenuated, subunit, inactivated, and nucleic acid vaccines (21, 22). Unfortunately, antigens generated by another expression organism tend to elicit an allergic reaction (23) and generally showed low immunogenicity, especially in resisting intracellular pathogens (21). Among many types of vaccine, DNA vaccine particularly aroused our interest, and many *in vivo* trials have demonstrated that DNA vaccine encoding one or multiple antigens can elicit an effectively humoral and cellular immune response against toxoplasmosis (24, 25). In addition, DNA vaccines showed obvious advantages over other types of vaccines, such as being stable, having high immunogenicity, being easy to produce and transport, and being susceptible to generating long-term immunity. Approved by the Food and Drug Administration (FDA) (26), the pVAX1 vector is characterized by massive replication in *Escherichia coli* and high expression in most mammalian cells. Furthermore, efficient delivery systems are extensively studied and used as the critical components in vaccine formulations to protect the antigens from undesirable degradation and evoke adequate immunity reactions (27). Currently, many synthesized delivery systems have been proved to be efficient in enhancing immune responses (28). As a biodegradable polymer extensively applied in biomaterials for bone tissue engineering (29), poly-lactic-*co*-glycolic acid (PLGA) has been licensed for applications in drugs and vaccines by the FDA (30, 31). In addition, chitosan also offers advantages in drug and vaccine delivery (32), due to its nontoxic, biocompatible, and biodegradable nature (33). Also due to its valuable properties, chitosan has been proved to be safe in parts of dietary applications and wound dressings (34, 35). Currently, both PLGA and chitosan were widely applied in *T. gondii* vaccine delivery (27, 36), but few studies reported its applications in DNA molecule delivery.

In the current study, we constructed a novel DNA vaccine encoding the *T. gondii* ribosomal P2 protein, named TgP2-pVAX1 plasmid. Then some attempts in combining TgP2-pVAX1 plasmids with PLGA and chitosan nanospheres were conducted to develop the nano DNA vaccine. BALB/c mice were double-immunized with synthesized PLGA or chitosan nanospheres, and the parasite burden was investigated to evaluate the immune protection of nano DNA vaccines. Our findings highlighted the importance of nanosphere-delivered DNA vaccines in eliciting potent protection against *T. gondii* infections.

## Materials and Methods

### Parasites, Cells, and Animals

Maintained in the Ministry of Education (MOE) Joint International Research Laboratory of Animal Health and Food Safety, College of Veterinary Medicine, Nanjing Agricultural University, Nanjing, PR China, tachyzoites of the highly pathogenic *T. gondii* RH strain (type I) were propagated *via* the BALB/c mice according to previous research (37).

Purchased from the Institute of Cell Biology, Chinese Academy Sciences, Shanghai, PR China, Human Embryonic Kidney 293-T (HEK 293-T) cells were cultured in Dulbecco’s Modified Eagle’s Medium (DMEM; Gibco, Carlsbad, CA, USA) containing 10% fetal bovine serum (FBS; Gibco, Carlsbad, CA, USA) and 1% double antibiotics (penicillin–streptomycin solution, Gibco, Carlsbad, CA, USA) at 37°C in a 5% CO_2_ atmosphere.

Obtained from the Model Animal Research Center of Nanjing University, Nanjing University, PR China, specific pathogen-free (SPF) BALB/c mice (18–22 g) and Sprague–Dawley rats (200–220 g) were strictly kept in an SPF environment. Supervised by the Animal Ethics Committee, Nanjing Agriculture University, China, all the animal-related operations were carried out in strict compliance with the requirements of the Ethics Procedures and Guidelines of the People’s Republic of China.

### Gene Cloning and Plasmid Construction

Total RNA Extraction kit (OMEGA Bio-Tek, Norcross, GA, USA) was used to isolate total RNA from 10^6^ tachyzoites of *T. gondii* according to the manufacturer’s protocol. By using a reverse transcription kit (Takara Biotechnology, Dalian, China), reverse transcription PCR (RT-PCR) was immediately employed to synthesize the cDNA. To amplify the complete open reading frame (ORF) of TgP2, primers were designed based on the conserved domain sequences (CDS) of TgP2 (GenBank: XM_002364187.2). Along with the Kozak translation initiation sequence and the restriction endonuclease sites (*Eco*RI and *Xho*I), primers, 5ʹ-CCG GAATTC GCCACC ATGGCAATGAAATACTTCGCTG-3ʹ (forward) and 5ʹ-CCG CTCGAG TTAGTCGAAGAGCGAGAAGCCC-3ʹ (reverse), were synthesized by Tsingke Biological Technology (Nanjing, China). Based on the instruction (Vazyme Biotech, Nanjing, China), PCR was performed in a volume of 50 μl, and the PCR amplification was conducted as follows: 1 cycle of 95°C for 5 min and then 35 cycles of 95°C for 30 s, 62.7°C for 30 s, and 72°C for 30 s. The final primer extension time was extended to 5 min at 72°C. The amplicons were detected using electrophoresis on a 1.0% agarose gel containing 0.01% ethidium bromide. The expected DNA bands were purified by Gel Extraction Kit (OMEGA Bio-Tek, Norcross, GA, USA), digested by *Eco*RI and *Xho*I restriction endonuclease (Takara Biotechnology, Dalian, China), and inserted into a linear pVAX1 vector (Invitrogen Biotechnology, Shanghai, China) by T4 DNA ligase (Takara Biotechnology, Dalian, China). The constructed plasmids were first determined by double restriction enzyme digestion and then sequenced by ABI PRISM™ 3730 XL DNA Analyzer (Applied Biosystems, Waltham, MA, USA). The correct plasmids were transferred into *E. coli* DH5α (Invitrogen Biotechnology, Shanghai, China). To obtain the plasmids in large quantity, *E. coli* DH5α carrying the correct plasmids was much duplicated in Luria Bertani (LB) medium containing 100 μg/ml of kanamycin monosulfate until the OD600 reached 0.6 (37°C and shaking at 180 rpm). The Endo-free DNA plasmid extraction kit (Vazyme Biotech, Nanjing, China) and ToxinSensor™ Chromogenic LAL Endotoxin Assay Kit (GeneScript, Piscataway, NJ, USA) was employed to obtain the endo-free DNA plasmids. Before being stored at −20°C, a nanodrop microvolume spectrophotometer (Thermo Scientific, Waltham, MA, USA) was used to determine the concentration of obtained plasmids.

### Expression Detection of Constructed Plasmids

To obtain the soluble tachyzoite antigens (STAg) of *T. gondii*, the previously described procedure was conducted with minor modifications (38). Briefly, the peritoneal lavage fluids were collected and passed through a 5-μm filtering membrane (Millipore, Billerica, MA, USA). After being centrifuged at 2,500 rpm for 5 min, 5 × 10^6^ tachyzoites were redissolved in 1,000 μl of phosphate-buffered saline (PBS). The protease and phosphatase inhibitor (Beyotime, Shanghai, China) were then added according to the instructions, and then tip sonication (Scientz Biotechnology, Ningbo, China) was carried out in a continuous mode for 2 s at 2 s (10 min in total) under an output power of 30 W. Before being at −20°C, the total amount of protein was conducted by bicinchoninic acid (BCA) assay (Thermo Scientific, Waltham, MA, USA).

To obtain the polyclonal antibody against *T. gondii* STAg, two Sprague–Dawley rats were first immunized with 500 μg of STAg mixed with complete Freund’s adjuvant (Sigma-Aldrich, St. Louis, MO, USA). Two weeks later, quartic immunizations were conducted using 500 μg of STAg mixed with incomplete Freund’s adjuvant (Sigma-Aldrich, St. Louis, MO, USA) at 1-week intervals. All immunizations were conducted subcutaneously in the back skin with multiple points. One week after the last injection, blood was harvested at the eye socket, and sera containing polyclonal antibodies were collected. Blank sera were also harvested from rats immunized with PBS using the same immunization strategy. All sera were kept at −20°C until use.

To determine the expression of constructed plasmids *in vitro*, 1 × 10^6^ HEK 293-T cells were cultured in a six-well plate (Costar, Cambridge, MA, USA) and were transfected with the endo-free plasmids using the Lipofectmine™ 3000 reagent (Invitrogen Biotechnology, Shanghai, China) according to the manufacturer’s guidelines. At 48-h stationary cultivation after transfection, the supernatants were discarded, and the cells were fixed with 1 ml of 4% paraformaldehyde for 12 h at 4°C after being rinsed three times with PBS. Then the cells were permeabilized with tris-buffered saline (TBS) containing 0.1% Triton X-100 (TBSx), blocked with TBSx containing 5% bovine serum albumin (BSA), and incubated with TBSx containing 5% BSA and serum against STAg (1:100 dilution). Rinsed 5 min in TBSx, the samples were incubated with CY3-conjugated anti-rat IgG (1:500 dilution, Sigma, Saint Louis, MO, USA) and subsequently with 4′,6-diamidino-2-phenylindole (DAPI) staining solution (500 μl/well, Beyotime, Shanghai, China). The images were immediately recorded by a Nikon A1 plus laser scanning confocal microscopy (Nikon Corporation, Tokyo, Japan).

To further confirm the expression of constructed plasmids *in vitro*, the transfected HEK 293-T cells were incubated at 37°C for 48 h. The cells were scraped into 500 μl of radioimmunoprecipitation assay (RIPA) lysis (Beyotime, Shanghai, China) containing protease and phosphatase inhibitor. Added with 10× loading buffer, HEK 293-T cell lysates were incubated at 95°C for 5 min and separated by 12% sodium dodecyl sulfate–polyacrylamide gel electrophoresis (SDS-PAGE). Cell lysates were transferred to methanol-activated polyvinylidene fluoride (PVDF) membrane (Millipore, Billerica, MA, USA) using a Semi-Dry Transfer system (Bio-Rad, Hercules, CA, USA). Membranes were blocked in TBS containing 0.05% (*v*/*v*) Tween 20 and 5% (*w*/*v*) non-fat skimmed milk powder on a rotary shaker with 70 rpm at 37°C for 2 h. Rinsed 5 min in TBST (TBS containing 0.05% (*v*/*v*) Tween 20), the membranes were incubated with polyclonal antibody against *T. gondii* STAg in TBST (1:100 dilution) at 4°C overnight with constant shaking. The membranes were subsequently incubated with horseradish peroxidase (HRP)-conjugated anti-rat IgG (1:8,000 dilution, eBioscience, San Diego, CA, USA) on a rotary shaker with 70 rpm at 37°C for 2 h. Rinsed 5 min in TBST, the blots were visualized by newly prepared 3,3′-diaminobenzidine (DAB; Sigma-Aldrich, St. Louis, MO, USA).

### Synthesis of Nano DNA Vaccines

To synthesize the PLGA nanospheres, the double emulsion solvent evaporation technique (*w*/*o*/*w*) was conducted based on the previous research with minor modifications (39, 40). To obtain the organic solution, 50 mg of PLGA (molecular weight (MW) 40,000–75,000 Da, LA/GA: 65/35, Sigma, Saint Louis, MO, USA) was weighed in a 50-ml centrifuge tube containing a magnetic stirring bar. Then 1 ml of dichloromethane (DCM; Sigma, Saint Louis, MO, USA) was added with the tube plugged and stirred until the complete dissolution of the polymer. Subsequently, polyvinyl alcohol (PVA; MW 31,000–75,000 Da, Sigma, Saint Louis, MO, USA) was dissolved in double-distilled water to prepare the 6% (*w*/*v*) PVA solution. Before use, the 6% PVA solution was passed through a 0.22-μm filtering membrane (Millipore, Billerica, MA, USA). At room temperature, 2 ml of 6% PVA solution was stirred using a magnetic stirrer (400 rpm), and 4 mg of endo-free DNA plasmids (concentration was 1 mg/ml) was dropwise dissolved by means of the Pipetman^®^ P1000L (Gilson, Shanghai, China). The aqueous solution was obtained after a subsequent mix on a vortex mixer for 5 min. The organic solution was dropped into the aqueous solution using a magnetic stirrer (400 rpm). Tip sonication (Scientz Biotechnology, Ningbo, China) was then carried out in a continuous mode for 2 s at 2 s (5 min in total) under an output power of 40 W under 4°C to synthesize the *w*/*o* emulsions. The *w*/*o* emulsions were dropped into 3 ml of 6% PVA solution using a magnetic stirrer (400 rpm). Tip sonication was again conducted with the same parameters to synthesize the *w*/*o*/*w* emulsions. The resulting *w*/*o*/*w* emulsions were then transferred into a 50-ml centrifuge tube using a magnetic stirrer (400 rpm) overnight to evaporate DCM. The *w*/*o*/*w* emulsions were centrifuged at 40,000 rpm for 15 min at 4°C, and PLGA nanospheres (precipitation) were collected in double-distilled water. In addition, the supernatant was also collected. After being passed through a 0.22-μm filtering membrane, the obtained PLGA nanospheres were then frozen. Once completely congealed, the nanospheres were transferred to a vacuum freeze-drier (Labconco, Kansas City, MO, USA) until completely freeze-dried. The freeze-dried PLGA nanospheres were stored at 4°C under sealed conditions until use.

To synthesize the chitosan nanospheres, the ionic gelation technique was conducted based on previous research with minor modifications (41). To make the chitosan solution, 100 mg of chitosan (MW 50–190 kDa, Sigma, Saint Louis, MO, USA) was dissolved in 50 ml of 1.0% (*v*/*v*) aqueous acetic acid solution and left in agitation until complete dissolution. Then the pH value was adjusted to 5.0 by NaOH solution. To prepare a 2 mg/ml sodium tripolyphosphate (TPP; Aladdin, Shanghai, China) solution, 20 mg of TPP was dissolved in 10 ml of double-distilled water and passed through a 0.22-μm filtering membrane (Millipore, Billerica, MA, USA). Chitosan solution measuring 10 ml was transferred into a 50-ml centrifuge tube using a magnetic stirrer (400 rpm) in an incubator at 30°C, and then 2 mg of endo-free DNA plasmids (concentration was 1 mg/ml) was added dropwise. Subsequently, 2 ml of TPP solution was dropped, and a 20-min constant stirring was conducted. Then tip sonication was then performed in a continuous mode for 2 s at 2 s (3 min in total) under an output power of 50 W. Centrifuged at 40,000 rpm for 15 min at 4°C, the chitosan nanospheres (precipitation) were collected in double-distilled water. The supernatant was also collected for further analysis. Passed through a 0.22-μm filtering membrane, the obtained CS nanospheres were then frozen. Once completely congealed, the nanospheres were transferred to the vacuum freeze-drier until completely freeze-dried. The freeze-dried CS nanospheres were stored at 4°C under sealed conditions until use.

### Characterization of Synthesized Nanospheres

To determine the loading capacity (LC) and encapsulation efficiency (EE) of synthesized nanospheres, the concentration of non-bound plasmids in the supernatant obtained in Section 2.4 was determined by the nanodrop microvolume spectrophotometer. LC and EE in PLGA and chitosan nanospheres were evaluated by applying formula (1) and formula (2), respectively.


(1) $$\text{LC (\%)}=\frac{\text{Total plasmid}-\text{Free plasmid concentration}\times \text{Volume of supernatant}}{\text{Weight of nanospheres}}\times 100\%$$

(2) $$\text{EE (\%)}=\frac{\text{Total plasmid}-\text{Free plasmid concentration}\times \text{Volume of supernatant}}{\text{Total plasmid}}\times 100\%$$

To investigate the morphological shape, the formulated nanospheres were sent to the College of Life Science, Nanjing Agriculture University, PR China, for scanning electron microscopy (SEM) observation (Hitachi SU8010, Hitachi Ltd., Tokyo, Japan). ImageJ software (version 1.8, NIH Image, Bethesda, MD, USA) was employed to characterize the average diameter of PLGA and chitosan nanospheres.

The release characteristics from PLGA and chitosan nanospheres *in vitro* were investigated in PBS (pH 7.4) as previously described (42). In brief, synthesized PLGA and chitosan nanospheres were dispersed in PBS (pH 7.4) at 37°C under mild shaking (180 rpm). At a 12-h interval, samples of the supernatant were collected by centrifugation and stored at −20°C. The settled nanospheres were resuspended in the original solution after each collection. After the last collection, the plasmid concentration in the samples was measured by a nanodrop microvolume spectrophotometer, and the cumulative release (CR) was calculated by applying formula (3).


(3) $$\text{CR (\%)}=\frac{\text{Total volume of supernatant}\times \text{Plasmid concentration}}{\text{Total loaded plasmid}}\times 100\%$$

To evaluate the toxicity of synthesized nanospheres, 35 BALB/c mice were randomly divided into seven groups (n = 5 mice): Blank (immunized with an equal volume of PBS), Control (immunized with pVAX1 plasmids), pVAX1/CS (immunized with pVAX1/CS nanospheres), pVAX1/PLGA (immunized with pVAX1/PLGA nanospheres), TgP2-pVAX1 (immunized with TgP2-pVAX1 plasmids), TgP2-pVAX1/CS (immunized with TgP2-pVAX1/CS nanospheres), and TgP2-pVAX1/PLGA (immunized with TgP2-pVAX1/PLGA nanospheres) groups. Each animal was injected intramuscularly with a dose containing 300 μg of plasmids, which was tripled in the immune dosage compared with the regular dosage. Three days later, a booster vaccination was conducted with the same dosage and strategy. Sera were harvested from the eye socket 1 day after treatment, and the levels of creatinine (Cr) in serum and the blood urea nitrogen (BUN) were evaluated by the sarcosine oxidase method (Solarbio, Beijing, China) and urease-indophenol method (Solarbio, Beijing, China). During the test period, their physical health and mental status were observed and recorded.

### Immunization and Challenging Schedules in Mice

To investigate the protective immunity of two synthesized nanospheres, BALB/c mice weighing 18–22 g (7 weeks old) were randomized into seven groups (n = 28 mice), all animals were intramuscularly immunized twice in the leg muscles with multipoint at 2-week intervals (**Table 1**). Blood samples were harvested through the eye socket before immunization, and the sera were separated and stored at −20°C until use. Blank or vaccinated animals were challenged with a lethal dose (10^3^ tachyzoites) of *T. gondii* RH strain intraperitoneally as indicated previously (43). Seven days after the challenge, infected animals were anesthetized and then sacrificed under the supervision of the Animal Ethics Committee, Nanjing Agriculture University, China, and the cardiac tissue was isolated and stored at −20°C until use.

**Table 1:** Immunization strategy in mice.

Group	Immunization strategy (each mouse)	Challenge strategy (each mouse)
Blank	Equal volume of PBS at weeks 0 and 2	10^3^ tachyzoites were challenged intraperitoneally at week 4
Control	100 μg of pVAX1 plasmid at weeks 0 and 2	
pVAX1/CS	pVAX1/CS nanospheres containing 100 μg of pVAX1 plasmid at weeks 0 and 2	
pVAX1/PLGA	pVAX1/PLGA nanospheres containing 100 μg of pVAX1 plasmid at weeks 0 and 2	
TgP2-pVAX1	100 μg of TgP2-pVAX1 plasmid at weeks 0 and 2	
TgP2-pVAX1/CS	TgP2-pVAX1/CS nanospheres containing 100 μg of TgP2-pVAX1 plasmid at weeks 0 and 2	
TgP2-pVAX1/PLGA	TgP2-pVAX1/PLGA nanospheres containing 100 μg of TgP2-pVAX1 plasmid at weeks 0 and 2	

### Antibody and Cytokine Investigation

To investigate the titers of *T. gondii*-specific serum antibody in the sera, ELISAs were conducted as described previously (44). Briefly, each well (96-well plates, Costar, Cambridge, MA, USA) was coated with 1 μg of STAg (dissolved in 100 μl of carbonate buffer pH 9.6) overnight at 4°C. After 5-min rinsing in TBST, each well was blocked with TBST containing 5% BSA (Sangon Biotech, Shanghai, China) at 37°C for 1 h. Sera from mice were diluted (1:100) in TBST containing 5% BSA and added into each well after being rinsed in TBST for 5 min. Incubated at 37°C for 1 h, each well of the 96-well plates was rinsed 5 min in TBST and incubated with HRP-conjugated anti-mouse IgG, IgG1, or IgG2a (1:8,000, eBioscience, San Diego, USA) at 37°C for 1 h. Finally, tetramethylbenzidine (TMB; Tiangen, Beijing, China) as substrate was used to evaluate the immunoreaction, and the reaction in each well was stopped by 100 μl of 2 M newly prepared H_2_SO_4_. The absorbance was determined at 450 nm by a microplate reader (Thermo Scientific, Waltham, MA, USA).

To determine cytokine secretions in animals’ sera, commercially available ELISA kits (Yifeixue Biotech, Nanjing, China) based on double antibody sandwich method were used to evaluate the concentrations of interferon-gamma (IFN-γ), interleukin (IL) 4 (IL-4), IL-10, and IL-17 referenced to known amounts of mouse recombinant cytokines.

### Flow Cytometry Analysis

At weeks 2 and 4 (before immunization or challenge), five mice from each group were euthanized, and the splenic lymphocytes were harvested by the lymphocyte separation kit (Solarbio, Beijing, China). To investigate the surface markers of dendritic cells (DCs), the separated lymphocytes were cultured in DMEM containing 10% FBS and 1% double antibiotics overnight. The non-adhering cells were discarded, and the attached cells were collected gently after being washed three times in PBS. Cells were then adjusted to 10^6^ cells in 100 μl of PBS and stained with anti-mouse CD11c-APC, CD86-FITC, and CD83-PE (eBioscience, San Diego, CA, USA) for 40 min at 4°C in the dark. After washing, centrifugation, and collection, cells were sorted by flow cytometry (Beckman Coulter Inc., Brea, CA, USA). Before cell sorting, the fluorescence compensation trial was performed to determine adequate compensation according to the instructions.

For the MHC molecules changes in DCs, the separated lymphocytes were directly adjusted to 10^6^ cells in 100 μl of PBS and stained with anti-mouse CD11c-PE, MHC-I-FITC, and MHC-II-APC (eBioscience, San Diego, CA, USA) under the same conditions mentioned above. Then the flow cytometry analysis was conducted using the same strategies described previously.

To analyze the proportion of CD4^+^ and CD8^+^ T lymphocytes subsets, 2 × 10^6^ splenic lymphocytes were equally dissolved in two tubes containing 100 μl of PBS. One tube was stained with anti-mouse CD3e-PE and CD4-FITC (eBioscience, San Diego, CA, USA) for CD4^+^ T lymphocyte subset detection, while another tube was stained with anti-mouse CD3e-PE and CD8-FITC (eBioscience, San Diego, CA, USA) for CD8^+^ T lymphocyte subset detection. The staining strategy and flow cytometry analysis were conducted using the same strategies described previously.

### Lymphocyte Proliferation Investigation

Three days after the booster immunization, three mice from each group were euthanized to separate the splenic lymphocytes using the same method described in Section 2.8. The splenic lymphocytes were adjusted to 10^5^ cells/well in a 96-well plate. The transfected cells described in Section 2.3 were scraped into 1 ml of sterile PBS, and then tip sonication was conducted to obtain the cell lysates in a continuous mode for 2 s at 2 s (10 min in total) under an output power of 30 W. After protein determination by BCA assay, 50 μg/well cell lysates was added into each well in a 96-well plate. The splenic lymphocytes with cell lysates were incubated at 37°C in a 5% CO_2_ atmosphere for 48 h. Cell Counting Kit 8 (CCK-8; Beyotime, Shanghai, China) measuring 10 μl was added according to the instructions. Incubated for 2 h, the plate was subsequently measured at 450 nm by a microplate reader to illustrate the lymphocyte proliferation.

### Parasite Burden in Mice

To demonstrate the parasite burden in mice, 30.0 mg of cardiac tissue obtained in Section 2.6 was lysed for genomic DNA extraction following the guidelines (OMEGA Bio-Tek, Norcross, Georgia, USA), and the DNA extracts were stored at −20°C until use. Absolute quantitative real-time PCR (qPCR) was carried out to investigate the 529-bp fragments in the extracts according to the published research (45). The plasmids containing the amplified 529-bp fragments were also constructed, and an online tool (http://cels.uri.edu/gsc/cndna.html) was employed to calculate the copy numbers of the plasmids. For qPCR amplification, 6.0 μl of 2× ChamQ Universal SYBR qPCR MasterMix (Vazyme, Nanjing, China), 0.24 μl of each primer, 1 μl of DNA extracts, and double-distilled water to make a final volume of 12.0 µl were involved in each reaction. Amplification for each reaction was performed on an Applied Biosystems 7500 (Life Technologies, Carlsbad, CA, USA) by 30-s incubation at 95°C, followed by 40 cycles of 10 s at 95°C and 30 s at 60°C. At the end of the reaction between 60°C and 95°C with an increment of 0.05°C/s, a melting curve analysis was performed. Before further analysis, the melting curve of each amplification was identified with one uniform peak as expected.

### Statistical Analysis

Conducted by GraphPad 6.0 software (GraphPad Prism, San Diego, CA, USA), a one-way ANOVA was employed to illustrate differences between groups. Pairwise comparisons among TgP2-pVAX1, TgP2-pVAX1/PLGA, and TgP2-pVAX1/CS group were estimated by ANOVA following Bonferroni’s correction. The results were considered as significant at *p* < 0.05 and represented as mean ± SD for each group. CytExpert software (version 2.3, Beckman Coulter Inc., Brea, CA, USA) was recruited to conduct the flow cytometric analysis.

## Results

### Plasmid Construction and Expression

**Figure 1:**
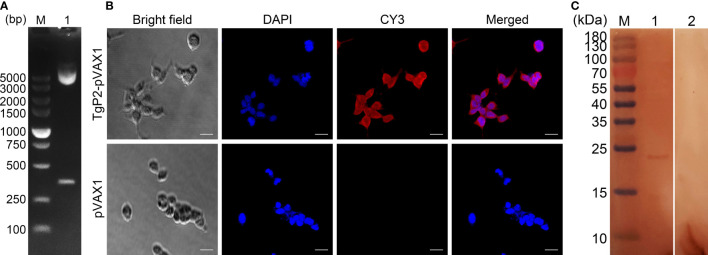
**(A)** Double digestion analysis of the recombinant plasmid TgP2-pVAX1. Line M, DL5000 marker; Line 1, double digestion with *Eco*RI and *Xho*I. **(B)** Immunofluorescence analysis of the expression of TgP2-pVAX1 in HEK 293-T cells. Bar represents 25 μm. **(C)** Western blotting of cell lysates probed with rat polyclonal antibodies against *Toxoplasma gondii* STAg. Cell lysates of HEK 293-T cell transfected with TgP2-pVAX1 (Line 1) and pVAX1 (Line 2) plasmids. Line M, MW marker proteins.

The TgP2-pVAX1 plasmid was successfully constructed as described previously. To verify the constructed plasmid, the double enzyme digestion was performed with *Eco*RI and *Xho*I, yielding two fragments of 354 and 2,966 bp, respectively (**Figure 1A**). The sequence results also indicated that the insert in the constructed plasmid was the ORF of TgP2. In summary, all these results proved that the TgP2-pVAX1 plasmid was correctly constructed.

The HEK 293-T eukaryotic cells were transfected with an endo-free TgP2-pVAX1 plasmid. As illustrated in **Figure 1B**, cells transfected with TgP2-pVAX1 plasmid showed specific red fluorescence, whereas cells transfected with pVAX1 plasmid did not display red fluorescence, indicating the expression of the TgP2 protein. The Western blotting results revealed a single band (approximately 24 kDa, **Figure 1C**), indicating an MW that is twice as expected (11.77 kDa). The pH value of resolving gel used in the current research was 8.8, and TgP2 has been proved to oligomerize at higher pH values (46). According to the protein sequence of TgP2, it lacks cysteines altogether. Thus, it is expected that the recombinant TgP2 protein should not exist in the disulfide bond, indicating the dimerization of TgP2 in resolving gel. Similar results have been reported in the previous study (18). In addition, no band was detected in pVAX1 transfected cells, and all these findings indicated that the recombinant TgP2 protein could be expressed by TgP2-pVAX1 in HEK 293-T cells.

### Characterization of Nanospheres

**Figure 2:**
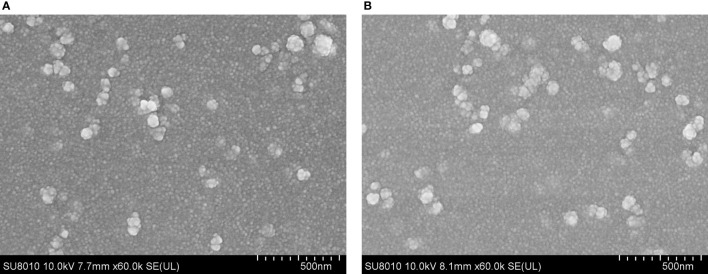
Scanning electron microscopy (SEM) images of TgP2-pVAX1 plasmids entrapped in poly-lactic-*co*-glycolic acid (PLGA) **(A)** and chitosan nanospheres **(B)**. By double emulsion solvent evaporation (*w*/*o*/*w*) and ionic gelation technique, PLGA and chitosan nanospheres were synthesized. After being completely freeze-dried, nanospheres were imagined at a magnification of ×30,000 (bar represents 500 nm).

After encapsulation of TgP2-pVAX1 plasmid in PLGA and chitosan nanospheres, the morphology of nanospheres was examined under SEM. As observed by SEM (**Figure 2**), the morphology of PLGA and chitosan nanospheres was both spherical in shape, with small round convex particles detected on its surface. According to five arbitrary nanospheres in the SEM images, the average diameter of TgP2-pVAX1/PLGA nanospheres synthesized by using 6% PVA was determined to be 112.31 ± 15.60 nm, while the mean diameter of TgP2-pVAX1/CS nanospheres formulated by using 2 mg/ml of TPP was 100.93 ± 12.36 nm. Based on five independent trials, the LC of PLGA and chitosan nanospheres was 1.91% and 4.16%, respectively, while the EE was 59.18% and 85.74%, respectively.

**Figure 3:**
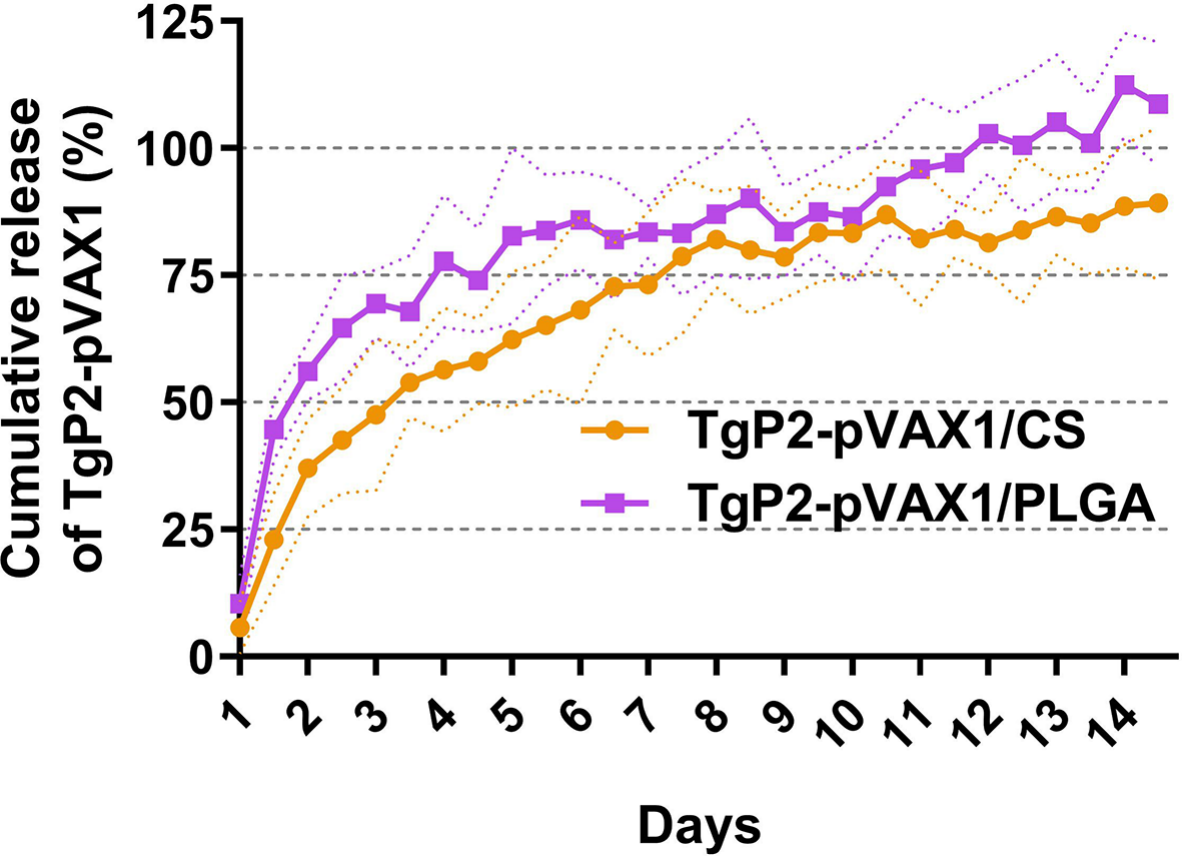
The release profile of poly-lactic-*co*-glycolic acid (PLGA) and chitosan nanosphere loaded with TgP2-pVAX1 plasmids *in vitro* over a 14-day period. Referenced to the blank PLGA or chitosan nanospheres, the total amount of TgP2-pVAX1 plasmids in the supernatant was determined by a nanodrop microvolume spectrophotometer. Three independent trials were carried out, and each sample was conducted once. Values are represented as mean ± SD (n = 3), and the dotted lines represent SD.

Referenced to the blank PLGA or chitosan nanospheres under the same circumstance, the release characteristics of TgP2-pVAX1/PLGA and TgP2-pVAX1/CS nanospheres were investigated. As demonstrated in **Figure 3**, the burst release of two types of nanospheres was both observed in the first 2 days, and the TgP2-pVAX1/CS nanospheres showed a steadier release profile as compared to the TgP2-pVAX1/PLGA nanospheres; 10.40% ± 5.67% of TgP2-pVAX1 were released from TgP2-pVAX1/PLGA nanospheres on the first day, while only 5.71% ± 5.17% plasmids were detected in the supernatant of TgP2-pVAX1/CS nanospheres on the first day. After the fourth day, the release profile of TgP2-pVAX1/PLGA nanospheres became flat, while the release curve became steep 6 days later (after the tenth day). However, the release curve of TgP2-pVAX1/CS nanospheres was slow-released at the first 7 days and became less steep after about 8 days. In addition, the CR of TgP2-pVAX1/PLGA nanospheres has crept up to over 100% on the twelfth day; such phenomenon may be caused by the existence of calculation error.

**Figure 4:**
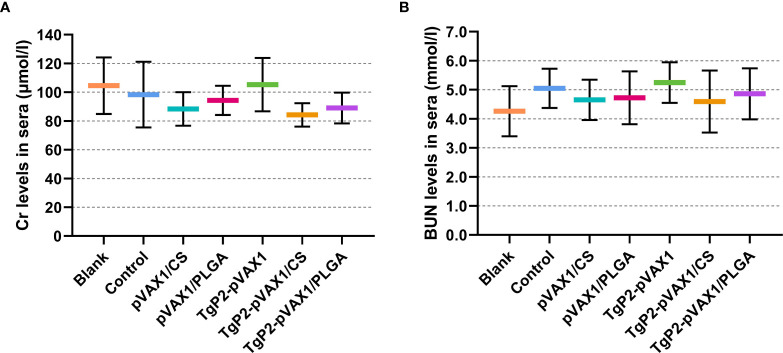
Toxicity analysis of TgP2-pVAX1 plasmid entrapped in poly-lactic-*co*-glycolic acid (PLGA) and chitosan nanospheres. Harvested from each group, sera were harvested, and the levels of Cr **(A)** and blood urea nitrogen (BUN) **(B)** were investigated based on the instructions of commercial kits. Each serum was conducted once, and significance was evaluated by one-way ANOVA followed by Dunnett’s test. Values among the TgP2-pVAX1, TgP2-pVAX1/PLGA, and TgP2-pVAX1/CS groups were pairwise compared by ANOVA following Bonferroni’s correction. Values are represented as mean ± SD (n = 5).

Based on the sarcosine oxidase and urease-indophenol method, the levels of Cr (**Figure 4A**) and BUN (**Figure 4B**) were analyzed to assess the toxicity of nanospheres. The levels of Cr and BUN remained at an acceptable range, and all groups were statistically similar (*p* > 0.05) to the blank or control group. In addition, no adverse reaction was observed in all animals, and no abnormal changes in the physical health and mental status. All the results indicated that the synthesized nanospheres were nontoxic.

### Antibody and Cytokine Determination

**Figure 5:**
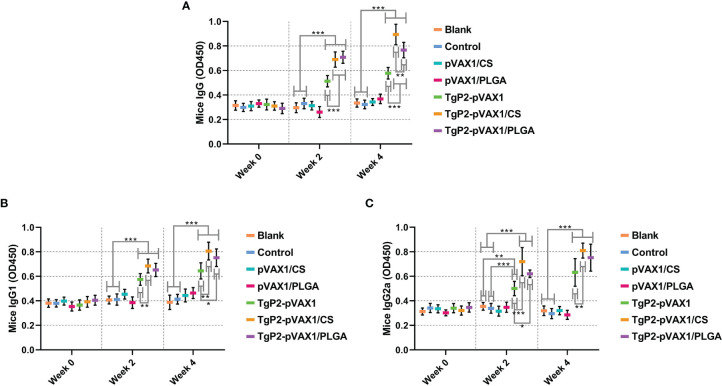
Antibody secretions of total IgG **(A)**, isotypes IgG1 **(B)**, and IgG2 **(C)** in the sera from immunized animals at weeks 0, 2, and 4. Each serum was conducted once, and significance was evaluated by one-way ANOVA followed by Dunnett’s test. Values among the TgP2-pVAX1, TgP2-pVAX1/PLGA, and TgP2-pVAX1/CS groups were pairwise compared by ANOVA following Bonferroni’s correction. Values are shown as the mean of the OD450 ± SD (n = 5). *p < 0.05, **p < 0.01, and ***p < 0.001 compared with blank or control group.

To determine whether the nanomaterial can promote the efficacy of TgP2-pVAX1 plasmids after multiple immunizations, mice were vaccinated with either TgP2-pVAX1 plasmids alone or nanospheres loaded with TgP2-pVAX1 plasmids one or two times. As expected, vaccinations of PLGA and chitosan nanospheres could induce higher levels of *T. gondii*-specific IgG (**Figure 5A**, *p* < 0.001) as compared with naked TgP2-pVAX1 plasmids at one and two immunizations. After the second immunization, animals immunized with TgP2-pVAX1/CS nanospheres could generate higher levels of IgG (*p* < 0.01) when compared to the animals immunized with TgP2-pVAX1/PLGA nanospheres. Furthermore, immunizations with the DNA vaccines entrapped in nanospheres as well as DNA vaccines alone could elicit higher levels of *T. gondii*-specific IgG1 (**Figure 5B**) and IgG2a (**Figure 5C**) antibody responses as compared with the blank or control group. When compared with naked TgP2-pVAX1 plasmids, immunizations with the TgP2-pVAX1/CS nanospheres could bolster statistically higher levels of IgG1 and IgG2a, regardless of immunization times. However, the TgP2-pVAX1/PLGA nanospheres could elicit significantly higher levels of IgG1 (*p* < 0.05) after the second immunizations and remarkable higher levels of IgG2a (*p* < 0.05) after the first immunization, when compared to the naked TgP2-pVAX1 plasmids.

**Figure 6:**
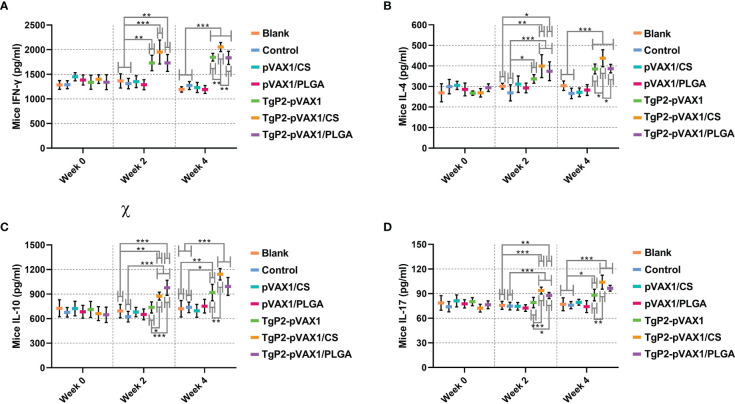
Cytokine secretions of IFN-γ **(A)**, IL-4 **(B)**, IL-10 **(C)**, and IL-17 **(D)** in animals’ sera at weeks 0, 2, and 4. Commercially available ELISA kits were used to determine the level of cytokines in the sera. Each serum was conducted once, and significance was evaluated by one-way ANOVA followed by Dunnett’s test. Values among the TgP2-pVAX1, TgP2-pVAX1/PLGA, and TgP2-pVAX1/CS groups were pairwise compared by ANOVA following Bonferroni’s correction. Values are shown as mean ± SD (n = 5). *p < 0.05, **p < 0.01, and ***p < 0.001 compared with blank or control group.

Following TgP2-pVAX1 plasmid immunization with or without nanospheres, sera of animals were harvested after the first and second immunizations to determine cytokine production. Immunizations with TgP2-pVAX1 plasmids or its nanomaterial encapsulations could enhance the production of cytokines IFN-γ (**Figure 6A**), IL-4 (**Figure 6B**), IL-10 (**Figure 6C**), and IL-17 (**Figure 6D**) when compared with blank or control group, regardless of the number of immunizations. In TgP2-pVAX1/CS group, animals receiving two immunizations could generate higher levels of IFN-γ, IL-4, IL-10, and IL-17 than those in the TgP2-pVAX1 group, while animals in TgP2-pVAX1/PLGA group receiving two immunizations could generate equal levels of four tested cytokines when compared with animals in TgP2-pVAX1 group (*p* > 0.05). Notably, animals in TgP2-pVAX1/CS group showed significantly higher levels of IFN-γ and IL-4 than those in the TgP2-pVAX1/PLGA group after the booster immunizations. All the obtained results suggested that TgP2-pVAX1 plasmids, TgP2-pVAX1/CS nanospheres, and TgP2-pVAX1/PLGA nanospheres could mediate the production of cytokines, and the concentrations in the sera were dependent on different nanospheres and the number of immunizations. In addition, the effects of PLGA or chitosan nanospheres in promoting the secretions of cytokines were also evaluated.

### Flow Cytometry Analysis in Splenic Dendritic Cells

**Figure 7:**
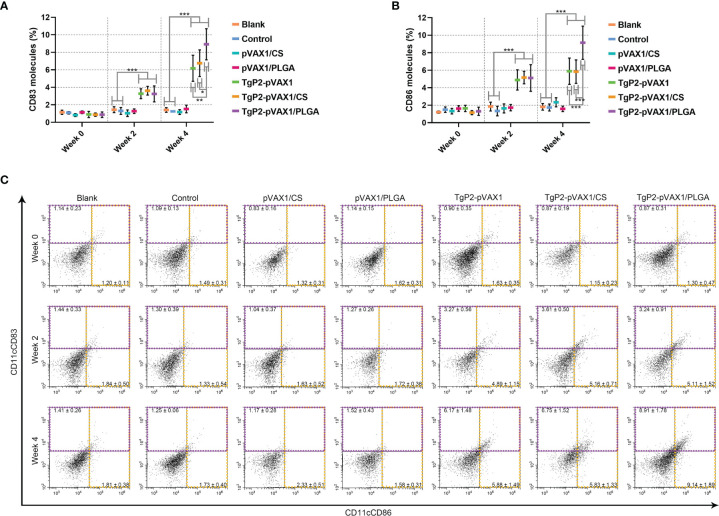
Flow cytometry analysis of dendritic cell (DC) maturation. Five animals in each group were sacrificed, and spleen lymphocytes from each animal were investigated. The bar graph shows the ratio of CD83 **(A)** and CD86 molecules **(B)** on splenic DCs, while the dot plots **(C)** show the percentages of CD11c^+^CD83^+^ and CD11c^+^CD86^+^ cells. Values are shown as mean ± SD (n = 5), and significance was evaluated by one-way ANOVA followed by Dunnett’s test. Values among the TgP2-pVAX1, TgP2-pVAX1/PLGA, and TgP2-pVAX1/CS groups were pairwise compared by ANOVA following Bonferroni’s correction. *p < 0.05, **p < 0.01, and ***p < 0.001 compared with blank or control group.

Splenic lymphocytes were collected from animals immunized once or twice with either naked TgP2-pVAX1 plasmids or nanospheres loaded with plasmids to determine the effect of nanosphere on DC maturation. As illustrated in **Figures 7A, C**, animals immunized with TgP2-pVAX1 plasmids, TgP2-pVAX1/CS nanospheres, or TgP2-pVAX1/PLGA nanospheres were detected with high expressions of CD83 on the surfaces of DCs (*p* < 0.001). Elicited by DNA vaccines and their nanospheres, high CD86 frequencies (*p* < 0.001) were also observed after the first or second immunization when compared with the blank or control group (**Figures 7B, C**). When compared with TgP2-pVAX1 plasmids, TgP2-pVAX1/PLGA nanospheres exhibited better effects in inducing CD83 and CD86 molecules according to the data analysis. Furthermore, statistical differences were observed between TgP2-pVAX1/CS and TgP2-pVAX1/PLGA group in promoting CD83 and CD86 molecules, with greater capacity of TgP2-pVAX1/PLGA nanospheres in inducing two tested molecules. All the obtained results indicated that the synthesized nanospheres as well as the TgP2-pVAX1 plasmids played a crucial role in inducing the CD83 and CD86 molecules of DCs, and the ability in enhancing surface molecule expression was dependent on the type of nanospheres and number of immunizations.

**Figure 8:**
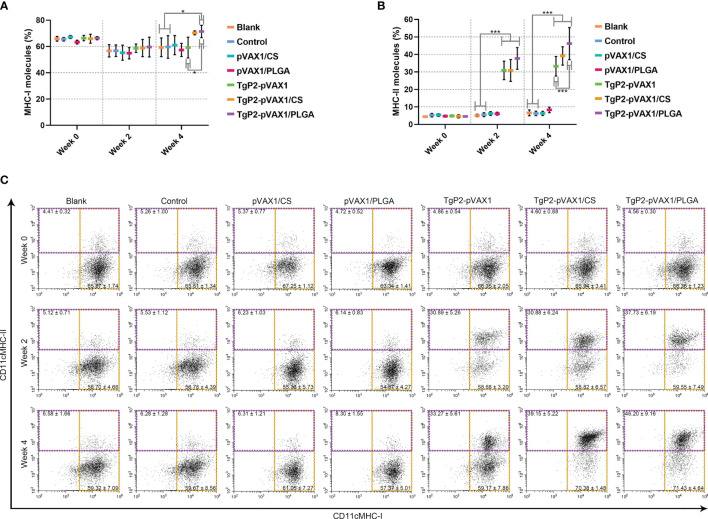
Flow cytometry analysis of MHC molecules on the surface of dendritic cells (DCs). Five animals in each group were sacrificed, and spleen lymphocytes from each animal were investigated. The bar graph shows the ratio of MHC-I **(A)** and MHC-II molecules **(B)** on splenic DCs, while the dot plots **(C)** show the percentages of CD11c^+^ MHC-I^+^ and CD11c^+^ MHC-II^+^ cells. Values are shown as mean ± SD (n = 5), and significance was evaluated by one-way ANOVA followed by Dunnett’s test. Values among the TgP2-pVAX1, TgP2-pVAX1/PLGA, and TgP2-pVAX1/CS groups were pairwise compared by ANOVA following Bonferroni’s correction. *p < 0.05 and ***p < 0.001 compared with blank or control group.

PLGA and chitosan nanospheres are known to be the delivery systems for antigens. To further evaluate the effects of TgP2-pVAX1/CS and TgP2-pVAX1/PLGA nanospheres on antigen presentation, murine lymphocytes were collected and analyzed by flow cytometry. Compared with the blank group (**Figures 8A, C**), TgP2-pVAX1/PLGA nanosphere vaccination induced higher levels of MHC-I molecules (*p* < 0.05) after booster immunization. However, the expressions of MHC-II molecules were significantly (*p* < 0.001) promoted by naked TgP2-pVAX1 plasmids or two types of nanospheres after the primary and booster immunizations (**Figures 8B, C**). In addition, TgP2-pVAX1/PLGA nanospheres exhibited higher capacity (*p* < 0.001) in eliciting MHC-II molecules after the booster immunizations, when compared with TgP2-pVAX1 plasmids. These obtained results indicated that the antigen presentation effects of DCs could be activated by TgP2-pVAX1 plasmids and their nanospheres, and an obvious promotion could be presented by the PLGA nanomaterial.

### Proliferation and Flow Cytometry Analysis in Splenic T Lymphocytes

**Figure 9:**
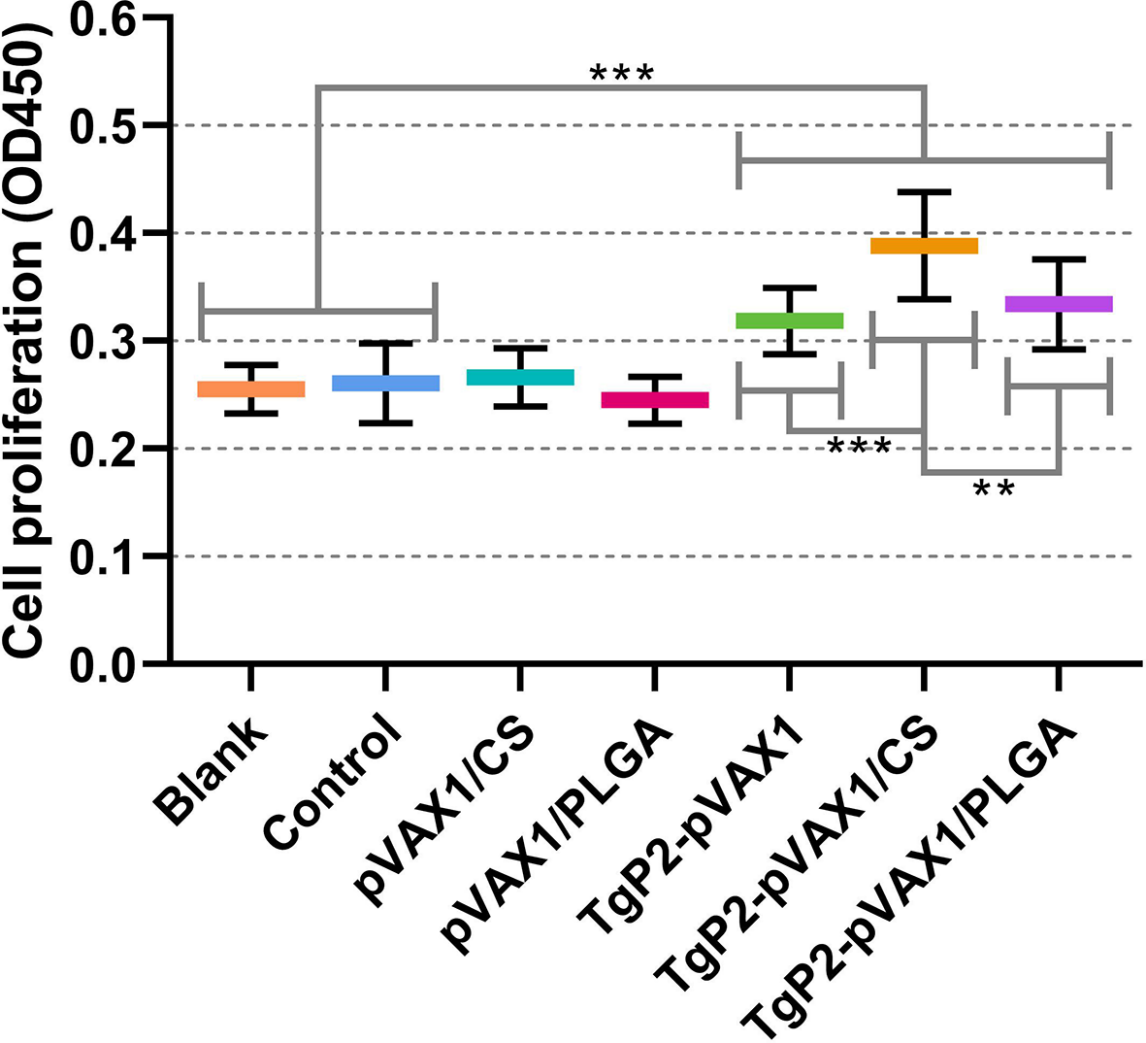
Splenocyte proliferation of animals. Three mice in each group were sacrificed, and spleen lymphocytes from each animal were investigated (n = 3). The obtained lymphocytes from each animal were divided into four parts and incubated with the HEK 293-T cell lysates transfected with phosphate-buffered saline (PBS) (Blank group), pVAX1 (Control group), and TgP2-pVAX1 (TgP2-pVAX1, TgP2-pVAX1/PLGA, and TgP2-pVAX1/PLGA groups). Each reaction was conducted once, and significance was estimated by one-way ANOVA followed by Dunnett’s test. Values among the TgP2-pVAX1, TgP2-pVAX1/PLGA, and TgP2-pVAX1/CS groups were pairwise compared by ANOVA following Bonferroni’s correction. **p < 0.01 and ***p < 0.001 compared with blank or control group.

The activated DCs are known to be highly effective stimulators that activate T lymphocytes. To determine whether the splenic T lymphocytes were effectively activated, splenic lymphocytes were separated 3 days after the booster immunization, and the lymphocyte proliferation was determined. As evaluated in **Figure 9**, animals immunized with TgP2-pVAX1 plasmids, TgP2-pVAX1/CS nanospheres, or TgP2-pVAX1/PLGA nanospheres could generate a significant proliferation when compared with the blank or control group. Compared with the naked TgP2-pVAX1 plasmids, TgP2-pVAX1/CS nanospheres could remarkably promote the proliferation of splenic lymphocytes (*p* < 0.001). Moreover, the significance could be also detected between animals immunized with TgP2-pVAX1/CS and TgP2-pVAX1/PLGA nanospheres (*p* < 0.01), indicating that the chitosan nanospheres were superior in inducing the proliferation of T lymphocytes.

**Figure 10:**
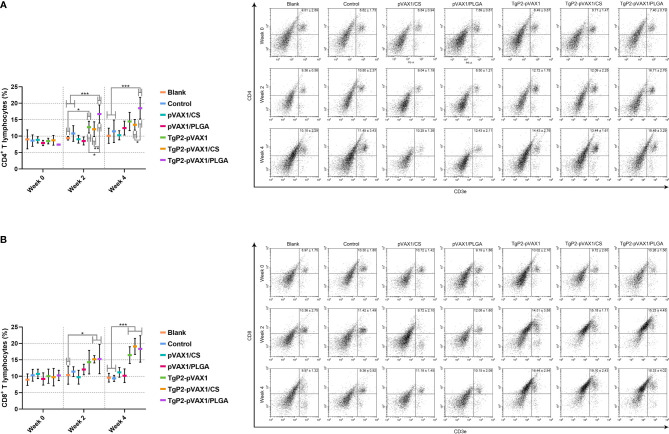
Flow cytometry analysis of CD4^+^ **(A)** and CD8^+^ T lymphocytes **(B)** in splenic lymphocytes. Five animals in each group were sacrificed, and spleen lymphocytes from each animal were investigated. Results were shown as mean ± SD (n = 5), and significance was estimated by one-way ANOVA followed by Dunnett’s test. Values among the TgP2-pVAX1, TgP2-pVAX1/PLGA, and TgP2-pVAX1/CS group were pairwise compared by ANOVA following Bonferroni’s correction. *p < 0.05, **p < 0.01, and ***p < 0.001 compared with blank or control group.

To demonstrate the PLGA and chitosan nanosphere effects on response inductions of T lymphocytes, splenic lymphocytes were harvested from animals immunized once or twice. Vaccination with TgP2-pVAX1 plasmids, TgP2-pVAX1/CS nanospheres, and TgP2-pVAX1/PLGA nanospheres induced higher levels of CD4+ (**Figure 10A**) and CD8+ T lymphocytes (**Figure 10B**) in animals’ spleen. Induced CD4^+^ T and CD8^+^ T lymphocytes were proportional to the number of immunizations, with animals receiving the booster immunizations showing better responses than animals immunized once. In addition, TgP2-pVAX1/PLGA nanospheres were better in CD4^+^ T lymphocyte enhancement when compared with the TgP2-pVAX1/CS nanospheres after the first and second immunization, and TgP2-pVAX1 plasmids, TgP2-pVAX1/CS nanospheres, and TgP2-pVAX1/PLGA nanospheres were equal in activating CD8^+^ T lymphocyte (*p* > 0.05).

### *Toxoplasma gondii* Burden in Animals

**Figure 11:**
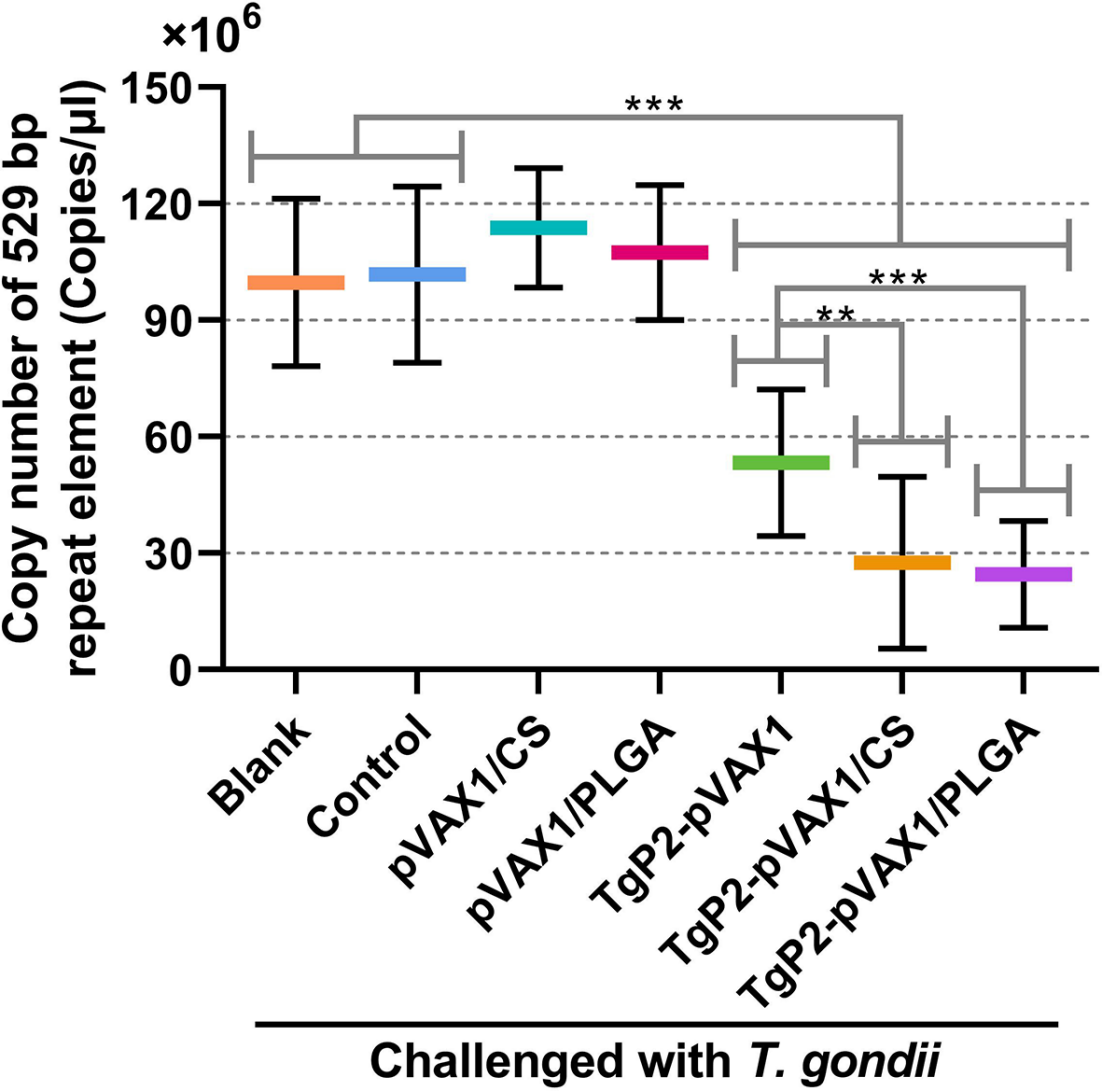
Copy number of 529-bp fragments in cardiac tissues from mice. Mice were intraperitoneally injected with 10^3^ tachyzoites of the highly virulent *Toxoplasma gondii* RH strain 1 week after the last immunization. One week later, five animals in each group were sacrificed, and cardiac tissues were harvested. Each DNA extract was run in triplicate, and the average value of each animal was calculated. Values are shown as mean ± SD (n = 5), and significance was evaluated by one-way ANOVA followed by Dunnett’s test. Values among the TgP2-pVAX1, TgP2-pVAX1/PLGA, and TgP2-pVAX1/CS group were pairwise compared by ANOVA following Bonferroni’s correction. **p < 0.01 and ***p < 0.001 compared with blank or control group.

To determine the effects of DNA vaccines and their nanospheres on improving protective efficacy, immunized animals were challenged with a lethal dose of *T. gondii* RH strain 2 weeks after the booster immunization. Parasite burden in heart tissue of challenged animals was investigated 7 days after challenge infection. As presented in **Figure 11**, animals immunized with the naked TgP2-pVAX1 plasmids and two types of nanospheres gained an advantage in resisting *T. gondii* over the unimmunized control (*p* < 0.001). In addition, compared with the naked TgP2-pVAX1 plasmids, both TgP2-pVAX1/CS (*p* < 0.001) and TgP2-pVAX1/PLGA nanospheres (*p* < 0.01) exhibited strong anti-*T. gondii* effect, and nearly two-fold differences of parasite burden were observed. All these obtained results suggested the importance of nanomaterials in inducing immune protection.

## Discussion

In the current study, the TgP2-pVAX1 plasmids were first constructed and then entrapped in PLGA or chitosan nanomaterials. The results suggested that TgP2-pVAX1/PLGA and TgP2-pVAX1/CS nanospheres with satisfactory release curves were spherical in shape and nontoxic to animals. In addition, evoked humoral immune responses, induced DCs and T lymphocytes, and inhabited *T. gondii* burden were demonstrated in animals immunized with TgP2-pVAX1/PLGA or TgP2-pVAX1/CS nanospheres. All these obtained results lent credit to the idea that TgP2-pVAX1 plasmids entrapped in PLGA or chitosan nanospheres could be a promising approach to resist the infections of *T. gondii* RH strain.

Successful vaccines require effective deliveries that can prevent antigens from degradation and induce robust humoral and cellular immunity (47, 48). As a promising vaccination method that induces host immune responses, DNA vaccination has emerged as a breakthrough in toxoplasmosis prevention (12, 49). PLGA nanospheres have an advantage as a DNA vaccine carrier, as they could protect the plasmids from undesirable degradation, and they could be efficiently absorbed by host cells for transcription (50, 51). However, the anionic property of PLGA nanospheres can result in poor mucoadhesiveness (52), and the delivery capacity is related to their size, surface properties, and even loaded antigens (36). As one of the nano-antimicrobial agents, chitosan is nontoxic and widely applied *in vitro* and *in vivo* to resist a wide variety of microorganisms (53, 54). It has been reported that chitosan could effectively activate cellular immunity *via* the cGAS-STING pathway (55). Furthermore, chitosan is a good option for gene delivery due to its cationic nature that can bind to nucleic acid and prevent it from degradation (56).

To enhance the immune efficiency, polymeric nanospheres have been widely used as the delivery vehicles for vaccines in previous studies (57, 58), and numerous methods have been constructed for nanosphere synthesis (30, 59). Based on the double emulsion solvent evaporation and ionic gelation technique, the PLGA and chitosan nanospheres were successfully formulated in the present study. Reported in Hela cells, 1,000-nm-sized nanospheres are observed with a lower absorption coefficient when compared with the 100-nm-sized nanospheres (60), and nanospheres with an average diameter less than 200 nm are easier to be internalized by DCs (61). The average diameter of PLGA and chitosan nanospheres synthesized in the current study was about 100 nm, implying a better absorption by host cells. As an important index to estimate the quality of formulated nanospheres, LC and EE can be affected by different preparation processes. According to a similar procedure, the EE of PLGA nanospheres was 57.5% in the previous study (62), while chitosan nanospheres whose EE and LC reached 92.8% and 63.7% were synthesized by Zheng et al. (63). The differences in LC and EE are likely to be driven by different procedures or the different nature of antigens, and further studies should focus on process optimization and enhancing the LC and EE.

Based on the SEM images, both TgP2-pVAX1/PLGA and TgP2-pVAX1/CS nanospheres had spherical morphology and appeared to be composed of many spherical structures. Such structures could prevent the obvious degradation of encapsulated antigens during the loading and release process. The PLGA nanospheres formulated in the present study showed a multiphasic release pattern, and such a pattern may be driven by porosity, nanosphere size, MW, and even the kind of antigens (64). Notably, the CR of PLGA nanospheres reached 100% on the twelfth day and increased in the following days. Such differences were likely due to measurement variability. TgP2-pVAX1/CS nanospheres exhibited a slow sustained release when compared with the TgP2-pVAX1/PLGA nanospheres. Negatively charged chitosan nanospheres were the cationic polymers that could bind to the surface of cells, leading to a constant residence (65). Such properties of chitosan nanospheres were beneficial for the antigens to induce robust immunity. Nanosphere applications in the vaccine or drug delivery are often limited by their manufacturing difficulty and toxicity (48). As the most commonly used solvent for PLGA, DCM was relatively volatile, easy to be removed, and widely used in biomaterial fabrications (66). However, a published paper questioned the safety of DCM in human beings (67). To fully remove the organic solvent and prolong the conservancy period, both TgP2-pVAX1/PLGA and TgP2-pVAX1/CS nanospheres were fully freeze-dried. As expected, the BUN and Cr levels in sera were similar, as well as the good mental shape in clinical observations, indicating that the synthesized nanospheres were nontoxic for host animals.

Humoral immunity is important in resisting *T. gondii*, and antibodies can mediate parasite phagocytosis and defense against the parasites and even induce the classical complement pathway to generate effective immunity (68). In the current study, massively secreted IgG was detected in animals immunized with naked plasmids and two types of nanospheres. It has been reported that subtype IgG1 antibody is associated with Th2-related immunity, while the Th1-related immunity is linked to subtype IgG2a (69). The observed titers of IgG2a were slightly higher than those of IgG1 after the first and second immunizations, suggesting the generation of a mixed Th1/Th2 immunity, but Th1-related immunity predominated. Compared with the DNA vaccines, PLGA and chitosan nanosphere-immunized animals could generate higher levels of IgG, IgG1, and IgG2a, indicating the immunity enhancement of two types of nanospheres. Such results were similar to the DNA vaccines against *T. gondii* previously reported (25, 70, 71).

Largely generated Th1-related cytokines play a crucial role in resisting *T. gondii* infection (68). As the major effector cytokine for host immunity against parasites, IFN-γ is generated by Th1 cells and plays an important role in activating host phagocytes and determining the fate of toxoplasmosis (72, 73). IFN-γ is involved in eradicating *T. gondii* through enhancing *T. gondii* tryptophan degradation (74), mediating nitric oxide (NO) synthesis (75), and activating immune-related GTPases (IRGs) (76). In the present study, significantly high levels of IFN-γ were observed in all vaccinated animals, and the Th2-related cytokines (IL-4 and IL-10) were also promoted. IL-4 plays a crucial role in promoting the secretions of IFN-γ at the late stage of parasite infections (77). Different from IL-4, IL-10 can inhabit severe immunopathology mediated by CD4^+^ T cells and limit excess inflammation to survive in animals (78). Cytokine IL-10 was enhanced in animals immunized with two types of nanospheres, indicating the regulation effects of Th2 cells. Generated by Th17 cells, IL-17 is identified as a potential tissue inflammatory regulator in resisting *T. gondii* (79, 80). Based on the published paper, IL-17 can participate in cellular infections by enhancing neutrophil recruitment (81). In the current research, we found the high levels of IL-17 secretion in nanosphere-immunized animals, indicating that the Th17 cells were also involved in resisting *T. gondii* infections.

As important antigen-presenting cells (APCs), DCs are of critical importance in activating innate and adaptive immunity (82). As the important checkpoint expressed on the surface of mature DCs, CD83 molecules play a critical role in the orchestration of immunity and the induction of resolution of inflammation (83), and early studies also indicated that CD83 molecules showed great influences on T-cell stimulation (84, 85). As important costimulatory molecules, CD86 molecules can bind to the CD28 molecules on T cells and provide the costimulatory signals to T cells that decreased the activation threshold of naïve T cells (86, 87). In addition, CD86 molecules are also critical in regulating antigen presentation (88). In the current study, all vaccine-immunized animals were observed with obviously enhanced CD83 molecules as well as the CD86 molecules, indicating that both TgP2-pVAX1/PLGA and TgP2-pVAX1/CS nanospheres could facilitate the maturation of DCs and increase the expression of partial costimulatory molecules. For antigen presentation to function, matured DCs can generate MHC-II molecules with the invariant-chain peptide on its surface, which could present antigens and mainly activated the CD4^+^ T cells (89, 90). In the current study, significantly high levels of MHC-II molecules were observed in animals immunized with naked plasmids and two types of synthesized nanospheres. Moreover, statistically promoted MHC-I molecules were also obtained in animals double-immunized with TgP2-pVAX1/PLGA and TgP2-pVAX1/CS nanospheres. Expressed in all karyocytes, MHC-I molecules are critical in endogenous antigen presentation, leading to the activation of CD8^+^ T cells (91). Through the cross-presentation pathway, MHC-I molecules can also transport exogenous antigen peptides to the surface of cells, resulting in the activation of CD8^+^ T cells (92). Furthermore, the published paper has proved that cytokine IFN-γ could upregulate the expression of MHC-I molecules through the JAK/STAT pathway (93). Thus, the up-expressed MHC-I molecules may be due to the secretion of IFN-γ. All obtained results indicated that both TgP2-pVAX1/PLGA and TgP2-pVAX1/CS nanospheres could promote DC maturation and induce slightly MHC-I-dependent and mainly MHC-II-dependent antigen presentation.

Generally, the activation of CD4^+^ T lymphocytes mainly requires two signals, MHC-II molecules and costimulatory molecules (94, 95), while CD8^+^ T lymphocytes can be activated by APCs or CD4^+^ T helper (Th) cells (96, 97). The activated T lymphocytes will undergo proliferation and differentiation (98), and T lymphocyte proliferation was regarded as the major characteristic in demonstrating immunity status (99). According to the current findings, all double-immunized animals could generate obvious proliferation, especially those immunized with TgP2-pVAX1/CS nanospheres. After proliferation, T lymphocytes then differentiate to different Th cells, and such a process often determines the type of immune response (100). The induced CD8^+^ T lymphocytes can differentiate into cytotoxic T lymphocytes (CTL) and then exhibit immunotoxicity to parasites (101). Mainly including Th1, Th2, Th17, and induced regulatory (iTreg) cells, activated CD4^+^ T lymphocytes can further activate the macrophages, recruit macrophages to the infection position, and generate cytokines (102). In addition, the published reports indicated that host *T. gondii* resistance is mainly conducted by natural killer (NK) cells for innate immunity and the CD4^+^ T lymphocytes for adaptive immunity (78). In the present study, a significantly increased proportion of CD4^+^ and CD8^+^ T lymphocytes was observed in nanosphere-immunized animals. These findings as well as the reports made it clear that TgP2-pVAX1/PLGA and TgP2-pVAX1/CS nanospheres played a critical role in the induction of host CD4^+^ and CD8^+^ T lymphocytes against toxoplasmosis.

To assess the immunoprotection of the synthesized nanospheres during the acute phase of *T. gondii* infection, the heart tissue was collected from each animal 7 days after infection, and the parasite burden was investigated. Nanosphere-vaccinated animals received lower *T. gondii* burden, indicating that a satisfactory immunoprotection could be elicited by TgP2-pVAX1/PLGA and TgP2-pVAX1/CS nanospheres. Currently, vaccines or drugs that could provide completely immunoprotection against toxoplasmosis have not been reported (12, 21), and our nanospheres loaded with TgP2-pVAX1 plasmids could be the promising vaccines in resisting *T. gondii* infections.

## Conclusion

In summary, this study suggests that nanosphere-delivered TgP2-pVAX1 plasmids were necessary for generating stronger immunoprotection against acute toxoplasmosis. The *in vivo* trials demonstrated that the nano DNA vaccines could induce strong humoral and cellular responses and significantly decrease the *T. gondii* burden in BALB/c mice, suggesting that nanomaterials were necessary for developing an effective nano DNA vaccine. Furthermore, our results also indicated that TgP2-pVAX1/CS nanospheres were similar to TgP2-pVAX1/PLGA nanospheres in resisting acute toxoplasmosis, according to the parasite burden. However, due to the intracellular nature of *T. gondii*, further investigations should optimize the LC of plasmids, further enhancing the cellular immune responses and verifying its efficiency on different animal models.

## Data Availability Statement

The original contributions presented in the study are included in the article/supplementary material. Further inquiries can be directed to the corresponding author.

## Ethics Statement

The animal study was reviewed and approved by the Animal Ethics Committee of Responsible Authority of the College of Veterinary Medicine, Nanjing Agricultural University, PR China.

## Author Contributions

ZY and XL designed the research. ZY, KH, and WC conducted the research. ZY and KH analyzed the data. ZY and MA wrote the manuscript. RY, LX, and XS participated in the revision of the manuscript. All authors contributed to the data interpretation and approved the final version of the manuscript.

## Funding

This research was funded by the Key Scientific and Technological Project of XPCC (2020AB025) and supported by College Students’ innovation and entrepreneurship training program (202110307025).

## Conflict of Interest

The authors declare that the research was conducted in the absence of any commercial or financial relationships that could be construed as a potential conflict of interest.

## Publisher’s Note

All claims expressed in this article are solely those of the authors and do not necessarily represent those of their affiliated organizations, or those of the publisher, the editors and the reviewers. Any product that may be evaluated in this article, or claim that may be made by its manufacturer, is not guaranteed or endorsed by the publisher.
